# Production and characterization of yeasts grown on media composed of spruce-derived sugars and protein hydrolysates from chicken by-products

**DOI:** 10.1186/s12934-020-1287-6

**Published:** 2020-02-03

**Authors:** David Lapeña, Gergely Kosa, Line D. Hansen, Liv T. Mydland, Volkmar Passoth, Svein J. Horn, Vincent G. H. Eijsink

**Affiliations:** 1 Faculty of Chemistry, Biotechnology and Food Science, Norwegian University of Life Sciences, P.O. Box 5003, 1432 Ås, Norway; 2 Department of Animal and Aquacultural Sciences, Norwegian University of Life Sciences, P.O. Box 5003, 1432 Ås, Norway; 3 Department of Molecular Sciences, Swedish University of Agricultural Sciences, P.O. Box 7015, 75007 Uppsala, Sweden

**Keywords:** Microbial protein, Yeast, Fermentation, Spruce, Protein hydrolysate, Feed, Aquaculture, Enzymatic hydrolysis

## Abstract

**Background:**

A possible future shortage of feed protein will force mankind to explore alternative protein sources that can replace conventional soymeal or fishmeal. Several large industrial organic side-streams could potentially be upgraded to feed protein using a fermentation process to generate single cell protein. Yeast is the most widely accepted microorganism for production of single cell protein, because of its superior nutritional quality and acceptability among consumers. Here, we have assessed the growth of four different yeasts, *Cyberlindnera jadinii*, *Wickerhamomyces anomalus*, *Blastobotrys adeninivorans* and Thermosacc^®^ Dry (*Saccharomyces cerevisiae*), on media composed of enzymatically saccharified sulfite-pulped spruce wood and hydrolysates of by-products from chicken, and we have characterized the resulting yeast biomass.

**Results:**

Generally, the yeast grew very well on the spruce- and chicken-based medium, with typical yields amounting to 0.4–0.5 g of cell dry weight and 0.2–0.3 g of protein per g of sugar. *B. adeninivorans* stood out as the most versatile yeast in terms of nutrient consumption and in this case yields were as high as 0.9 g cells and 0.5 g protein per g of sugar. The next best performing yeast in terms of yield was *W. anomalus* with up to 0.6 g cells and 0.3 g protein per g sugar. Comparative compositional analyses of the yeasts revealed favorable amino acid profiles that were similar to the profiles of soymeal, and even more so, fish meal, especially for essential amino acids.

**Conclusions:**

The efficient conversion of industrial biomass streams to yeast biomass demonstrated in this study opens new avenues towards better valorization of these streams and development of sustainable feed ingredients. Furthermore, we conclude that production of *W. anomalus* or *B. adeninivorans* on this promising renewable medium may be potentially more efficient than production of the well-known feed ingredient *C. jadinii*. Further research should focus on medium optimization, development of semi-continuous and continues fermentation protocols and exploration of downstream processing methods that are beneficial for the nutritional values of the yeast for animal feed.

## Background

The world´s population is projected to reach about 9.7 billion people in 2050 [1]. Such a population would need 1250 million tons of meat and dairy products per year to meet the demand for animal-derived protein assuming current consumption levels [2]. This growing demand will force mankind to search for alternative protein sources that can replace or supplement plant proteins that are currently used as animal feed. Of note, conventional plant proteins tend to be inefficiently converted: approximately 6 kg of plant protein is needed to produce 1 kg of meat protein [3]. Increasing meat production to match global demand is ultimately not sustainable [4].

Aquaculture, which currently contributes 17% of the global intake of animal protein [5], appears to be a possible solution, in part because of better feed conversion rates [6]. Fish meal and plant-based proteins are the currently preferred protein sources for many aquaculture species. However, increased use of fish meal is not sustainable since it is based on catch of wild fish stocks [7]. Plant-based proteins such as soybean protein require the use of arable land, raising potential ethical conflicts between food and feed production [8, 9]. Combined with the increasing demand for fish feed protein, these considerations show that other sources of protein must be identified and developed.

One solution for this challenge is to use microbial proteins, also known as single cell protein (SCP), produced by fungi, algae or bacteria. Yeasts are among the preferred candidates due to their rapid growth and high protein content, a low risk of contamination, and ease of harvesting due to their cell size and flocculation abilities [2, 9]. Yeasts are considered a well-balanced source of amino acids and can provide vitamins (mainly the B group) [10]. They also contain lower amounts of nucleic acids (5–12%) than bacteria (8–14%), which is beneficial for a human food or animal feed ingredient [2, 10]. Additionally, it has been shown that certain yeasts may have positive health effects in pigs [11], poultry [12] and fish [13], possibly as a result of the presence of bioactive and immunostimulating compounds such as ß-glucans and α-mannan.

The production of yeast biomass as a source of SCP should be cheap and environmentally friendly in order to replace the aforementioned unsustainable feed ingredients for the production of meat. Therefore, it is important to find yeast strains with optimal properties and to develop high quality, cheap and sustainable fermentation media. It has been estimated that in yeast SCP production, 62% of the total product cost comes from the raw materials used for fermentation [14]. Yeasts can convert readily available and low-cost industrial organic by-products into high quality protein and lipids for animal feed and even for human consumption [10, 15]. Hydrolyzed protein-rich by-products from food production, such as meat and fish residues, may be utilized as an alternative to inorganic nitrogen sources that are commonly used for fermenting yeasts Non-edible residues produced from agricultural and forestry industries, such as saw dust or straw, can be utilized as alternative carbon sources. Since such side-streams are rich in cellulose, hemicellulose and lignin and since yeasts do not have enzymes for efficiently processing these polymers, the use of these raw materials requires an enzyme pre-treatment to produce sugars that can be assimilated. Recently, the Norwegian company Borregaard developed a pretreatment technology for lignocellulosic biomass, which includes a sulfite cooking step that solubilizes lignin and washes out most of the hemicellulose, leaving a relatively clean cellulose fraction. After this process, called BALI, for Borregaard Advanced Lignin™ [16], modern cellulase cocktails can efficiently convert cellulose and hemicellulose into soluble hexoses and pentoses [17, 18].

The aim of this study was to use a medium consisting of sugars produced through enzymatic hydrolysis of lignocellulosic biomass [16, 19] and enzymatically hydrolyzed chicken by-products [20] to produce SCP in the form of yeast. Four different yeast strains were tested: *Cyberlindnera jadinii* (anamorph name *Candida utilis*), *Wickerhamomyces anomalus*, *Blastobotrys adeninivorans* (synonym *Arxula adeninivorans*) and Thermosacc^®^ Dry. We carried out a preliminary screening of growth in microtiter plates, where ten different growth media were tested. The best two media were then tested in batch fermentations using benchtop fermenters, where concentrations of cells, substrates, side-products and yeast protein were monitored. We also characterized and compared the four different yeast biomasses generated after the batch fermentations, assessing properties such as amino acid composition, and the content of nucleic acids, minerals, lipids, carbohydrates and ash. Finally, we assessed the composition of the yeast biomass with Fourier Transform Infrared Spectroscopy (FTIR).

## Methods

### Materials

Fresh chicken by-products (heart, liver and digestive tract) were provided by Nortura Hærland (Hærland, Norway) and kept at − 20 °C until further use. Prior to the enzymatic hydrolysis reactions, samples were thawed and minced with a BIRO^®^ MODEL 6642 feed grinder (Marblehead, Ohio, USA). The chicken by-products contained 30.12 ± 0.50% dry matter, including 15.10 ± 1.20% protein, 4.47 ± 0.29% ash and 6.91 ± 0.55% lipids [20]. Enzymatic hydrolysates of BALI™ pretreated spruce wood (*Picea abies*) were kindly provided by Borregaard AS (Sarpsborg, Norway) [16]. The raw material used in the pulping process was chipped spruce with chip sizes of up to 4.5 × 4.5 × 0.8 cm^3^. The carbohydrate composition of the spruce hydrolysate is shown in Additional file 1: Table S1. Yeast extract, meat peptone, yeast nitrogen base w/o amino acids and w/o ammonium sulfate, glucose, cellobiose, xylose, lactic acid, acetic acid, sulfuric acid, hydrogen chloride, sodium hydroxide, ninhydrin, glycine and stannous chloride were purchased from Sigma-Aldrich (Missouri, USA). Ammonium sulfate was purchased from VWR (Pennsylvania, USA), and urea was kindly provided by Yara International ASA (Oslo, Norway). Kjeltabs for Kjeldahl analysis were purchased from Thomson and Capper Ltd. (Cheshire, UK).

### Enzymatic hydrolysis of chicken by-products

15 kg (wet weight) minced chicken by-products were mixed with 15 L of water in 30 L Einar hydrolysis reactors (Belach Bioteknik, Skogås, Stockholm, Sweden), resulting in a dry-matter concentration of 15%. The enzymatic hydrolysis of the chicken by-products was carried out using 0.5% (weight of the enzyme powder/weight of wet chicken by-products) papain from *Carica papaya*, (≥ 3 U/mg; Merck, Darmstadt, Germany) at 60 °C and 50 rpm without pH adjustment and using slow heating to 60 °C, as described previously [20]. The hydrolysates were removed from the hydrolysis tanks after 2 h and were filtered through a sieve of 0.85 mm Ø in order to remove insoluble particles. Subsequently, the hydrolysates were cooled down to 4 °C and stored overnight, which led to accumulation of lipids on the top of the hydrolysate. The liquid fraction was centrifuged in a Beckman Coulter Avanti J-26S XP centrifuge (Indianapolis, Indiana, USA) at 4 °C and 10.000 g for 10 min. Finally, the chicken by-products hydrolysates (CH) were filtered using a sieve of 75 µm Ø and stored at − 20 °C until use. Due to the large hydrolysis volume (15 kg raw material and 15 kg water), the inactivation of proteolytic enzymes was not carried out directly after hydrolysis but by autoclaving of specific aliquot volumes used when preparing fermentation media.

### Growth experiments

#### Microtiter plates

*Cyberlindnera jadinii* LYCC 7549, Thermosacc^®^ Dry (*Saccharomyces cerevisiae*) (Lallemand Yeast Culture Collection, Montreal, Canada), *W. anomalus* CBS100487 (Strain collection of the Swedish University of Agricultural Sciences, Uppsala, Sweden, internal strain number J121) and *B. adeninivorans* LS3 (Swedish University of Agricultural Sciences, Uppsala, Sweden), were stored in cryovials containing 20% (v/v) glycerol at − 80 °C. Ten different media were tested for growth: yeast nitrogen base without amino acids and with ammonium sulfate plus glucose (YNBAS + G), yeast nitrogen base without amino acids and with ammonium sulfate plus BALI™ hydrolysate (YNBAS + B), yeast nitrogen base without amino acids and with urea plus BALI™ hydrolysate (YNBU + B), yeast extract and meat peptone plus glucose (YP + G), yeast extract and meat peptone plus BALI™ hydrolysate (YP + B), chicken by-products hydrolysate plus glucose (CH + G), chicken by-products hydrolysate plus BALI™ hydrolysate (CH + B), chicken by-products hydrolysate (CH) and BALI™ hydrolysate (B). The nitrogen content (5.86 g/L; based on the nitrogen content of YP, containing 20 g/L yeast extract and 30 g/L meat peptone, as measured by Kjeldahl) and intended glucose content (50 g/L) were identical in all media, except in the control media containing only sugar (B) or only protein (CH). Note that the BALI hydrolysate contains an extra 16 g/L of carbohydrates per 50 g of glucose (Additional file 1: Table S1). All medium components were individually sterilized by autoclaving at 121 °C for 20 min and then mixed to obtain media with the desired compositions. Overnight pre-cultures were prepared by adding 200 µL of a seed culture to 25 mL of the to-be-tested medium in a 250 mL baffled shake flask, followed by incubation at 30 °C, 220 rpm for approx. 16 h.

The four yeast strains were grown in the Duetz-microtiter plate system (Duetz-MTPS) (Enzyscreen, Heemstede, The Netherlands), consisting of 24-square polypropylene deep well plates (11 mL), sandwich covers and cover clamps, which were mounted in a shaker (Infors HT Shaker Minitron, Bottmingen, Switzerland). Autoclaved and dried microtiter plates were filled with 2.5 mL of sterile liquid medium. The initial pH was adjusted to 5.0 using 1 M NaOH or HCl. Media were inoculated with the overnight pre-cultures to obtain an initial OD of 0.5, as measured at 595 nm with a UV/VIS spectrophotometer (Hitachi U1900, Tokyo, Japan). The plates were incubated at 30 °C at 450 rpm and samples were taken at 8 h, 16 h and 24 h, for the measurement of cell dry weight (CDW), free amino nitrogen and pH. Note that experiments were set up with multiple wells per condition and that each well was only sampled once for the measurement of cell dry weight (CDW), free amino nitrogen and pH. These experiments were performed in triplicates.

#### Batch fermentations

The bioreactor cultivations were performed in 2.5 L total volume glass fermenters (Minifors, Infors, Bottmingen, Switzerland) with working volumes of 1.5 L and equipped with two 6-bladed Rushton impellers, using YP + G (i.e. 30 g/L meat peptone, 20 g/L yeast extract and 50 g/L glucose) and CH + B (i.e. the “chicken + spruce” medium). YP or CH were autoclaved at 121 °C for 15 min in the bioreactors. Glucose and BALI™ hydrolysate were autoclaved separately. Overnight pre-cultures were prepared by adding 1 mL of a seed culture [80% (v/v) of an overnight culture on standard YPD (Sigma-Aldrich, Missouri, USA), 20% (v/v) glycerol, stored at − 80 °C] into 250 mL of YP + G or CH + B medium in 2 L baffled shake flasks, followed by incubation at 30 °C, 220 rpm for approx. 16 h. The bioreactors were inoculated with 30 mL overnight preculture (2% (v/v) and each fermentation was run in duplicates. The temperature for all cultivations was 30 °C. The pH was monitored with a pH probe (Mettler Toledo, Greifensee, Switzerland) and was kept at 5.0 by automatic addition of 1 M NaOH or 5 M H_2_SO_4_. The DO was maintained at approximately 30% saturation (± 5%) and regulated by manual adjustment of the stirrer speed (300–1250 rpm). Cultures were aerated through a sparger at an initial rate of 1.5 L/min (1 VVM), which was increased to up to 3 L/min (2 VVM) during the fermentation to maintain DO. Exhaust CO_2_ and O_2_ analysis was performed with a FerMac 368 off-gas analyzer (Electrolab Biotech, Tewkesbury, UK). Foam was controlled via a foam sensor with five times diluted Glanapon DB 870 antifoam (Busetti, Vienna, Austria). Fermentation data were recorded using IRIS process control software (Infors). During the fermentation, samples were aseptically taken every two hours for analysis of the culture supernatant and the yeast biomass.

### Analytical methods

#### Cell dry weight (CDW)

Fermentation broth samples (25 ml from bioreactors and 2 ml from microplates) were centrifuged at 4700 g (25 mL) or 10,000 g (2 mL) for 5 min at 4 °C and the supernatant was collected for further analyses (sugars, organic acids, ethanol, free amino nitrogen, protein). Then, the yeast cells were washed twice with cold distilled water, frozen at − 20 C and then freeze–dried using an Alpha 2–4 LDplus (Martin Christ, Osterode am Harz, Germany), at − 60 °C and 0.01 mbar vacuum for 48 h. After determining their weight, to calculate CDW, the dried cells were used for analysis of protein content, amino acids, nucleic acids, lipids, fibers, minerals and ash, and were also analyzed by FTIR spectroscopy.

#### Monosaccharides, organic acids and ethanol

Monosaccharides (d-glucose, d-xylose), organic acids (lactic acid, acetic acid) and ethanol present in the fermentation broth were analyzed by high performance liquid chromatography (HPLC) with refractive index detection. The samples were diluted 10 times with distilled water and 200 µL were vacuum-filtered through 96 well filter plates (0.45 μm). Samples were separated on a Rezex ROA-organic acid H+, 300 × 7.8 mm^2^ (Phenomenex, Torrance, CA, USA) analytical column fitted with a cation–H cartridge guard column, using a column temperature of 65 °C, 5 mM H_2_SO_4_ as eluent and a flow rate of 0.6 mL/min. Additionally, monosaccharides were analyzed by High-Performance Anion-Exchange Chromatography (HPAEC) using a Dionex ICS 3000 system (Dionex, Sunnyvale, CA, USA) equipped with a CarboPac PA1 column operated at 30 °C, and with pulsed amperometric detection (PAD), where d-xylose, l-arabinose, d-mannose and d-galactose could be quantified. Monosaccharides were eluted isocratically at a flow rate of 0.25 mL/min using 1 mM KOH, generated with an eluent generator. HPLC data were collected and analyzed using Chromeleon 7.0.

#### Free amino nitrogen

A ninhydrin-based assay was performed in order to follow the consumption of free amino nitrogen during fermentations [21]. Ninhydrin reacts with free alpha amino groups resulting in a blue product that can be colorimetrically quantified by measuring the absorbance at 575 nm. Glycine was used to produce a standard curve with known free amino nitrogen content (0.04–0.4 g/L nitrogen). 10 µL (diluted, if appropriate) samples were added to 100 µL of pH 5.5 acetate buffered ninhydrin reagent (containing 25 µL/mL SnCl_2_), mixed, and incubated at 100 °C for 10 min. The assay was performed in microplate format (two replicates) and absorbance was measured with a Synergy H4 Hybrid Multi-Mode Microplate Reader (BioTek, Winooski, Vermont, U.S.)

#### Protein content

Total nitrogen was measured according to the Kjeldahl method (European Commission [EC] regulation No: 152/2009, pp 15–19) using a Kjeltec TM 8400 (FOSS, Tecator, Höganäs, Sweden) after acid digestion in an autodigestor (FOSS, Tecator, Höganäs, Sweden). The protein content of samples was estimated by multiplying total nitrogen by a factor of 6.25.

#### Amino acids

Analysis of the content of amino acids (except tryptophan) in freeze–dried yeast was performed according to EC regulation No: 152/2009 (pp. 23–32) using a Biochrom 30 amino acid analyzer (Biochrom Ltd., Cambridge, UK). Tryptophan was analyzed according to EC regulation No: 152/2009 (pp. 32–37) using a Dionex Ultimate 3000 HPLC system (Dionex Softron GmbH, Germering, Germany) connected to a RF-535 fluorescence detector (Shimadzu., Kyoto, Japan). All amino acids were quantified by using external standards (Dionex Ltd., Surrey, UK).

#### Nucleic Acids

The nucleic acid content in yeast biomass was determined spectrophotometrically by the diphenylamine assay (DNA) and the orcinol assay (RNA) after extraction with diluted perchloric acid [22]. Before extraction, 50 mg of freeze–dried yeast cells were washed with 1.5 ml 0.9% saline solution (cold), and then with 1.5 ml 0.2 M HClO_4_ (cold). Subsequently, 1.5 ml of 0.5 M HClO_4_ was added, and the cells were incubated at 70 °C for 15 min, centrifuged, and the supernatant was saved. Another 1.5 ml 0.5 M HClO_4_ was the added to the cells, followed by mixing, another incubation at 70 °C for 15 min, and centrifugation. The supernatants were combined and diluted to 5.0 mL with 0.5 M HClO_4_. The HClO_4_-extracts from the yeast cells and DNA standards (calf thymus DNA; Sigma D4522) were mixed with diphenylamine reagent 1:1 (v:v) (stock solution of 1.5 g diphenylamine, 100 mL glacial acetic acid, 1.5 mL concentrated sulfuric acid and 1 mL acetaldehyde solution) in 96 well plates, incubated at 30 °C over night, and absorbance was read at 600 nm on a Spectramax M2^e^ microplate reader (Molecular Devices, LLC, San Jose, CA, USA). HClO_4_-extracts from the yeast cells and RNA standards (RNA from baker’s yeast; Sigma R6750) were mixed with a H_2_SO_4_/H_2_O solution (v/v; 85/15) in 96 well plates, and incubated at 40 °C for 24 h. The orcinol reagent (stock solution of 0.35 mL 6% (w/v) orcinol to 5 mL concentrated HCl) was then added, and the plates were incubated with gentle shaking at 100 °C for 30 min, after which sbsorbance at 500 nm was read on a Spectramax M2^e^ microplate reader (Molecular Devices).

#### Lipids

The total lipid content of the freeze–dried yeast biomass was determined using accelerated solvent extraction [23]. The extraction was carried out at 125 °C and 1500 psi with a mixture of 70% petroleum ether − 30% acetone in a Dionex ASE 350 accelerated solvent extractor (Dionex, Sunnyvale, CA, USA). Then, the solvent was placed in a collection glass which was immersed in a 60 °C water bath for evaporation under N_2_ pressure. After 10 min, only lipids remained in the collection glass, and they were placed in a vacuum drier at 70 °C for 30 min. Finally, the samples were placed in a desiccator, and lipids were weighed.

#### Minerals and ash

The mineral content of freeze–dried yeast biomass was analyzed by inductively coupled plasma mass spectrometry (ICP-MS) (Agilent 8800 QQQ, Santa Clara, California, USA). Samples were decomposed by 65% HNO_3_ in a high performance microwave reactor (UltraClave, MLS, Milestone, Sorisole, Italy) [24]. For halides (anions), the samples were decomposed using concentrated 25% (w/w) tetramethylammonium hydroxide. The mineral analyses were validated using certified reference materials NCS DC73349 (National Analysis Center for Iron and Steel, Beijing, China) and CRM GBW07603 (National Research Centre for CRM, Beijing, China). The ash content of freeze–dried yeast biomass was determined according to the technical report NREL/TP-510-42622 from the National Renewable Energy Laboratory [25].

#### Fourier Transform Infrared (FTIR) Spectroscopy

FTIR analysis of freeze–dried yeast biomass was performed with an Agilent 5500 Series FTIR Spectrometer (Agilent, Santa Clara, US) using a single-bounce type IIA diamond crystal attenuated total reflectance (ATR) accessory with sample press. Approximately 10 mg freeze–dried yeast samples were measured in the spectral range of 4000–650 cm^−1^ with a resolution of 8 cm^−1^ and 32 scans. Background spectra (empty crystal) were measured before each sample and used for correction. The diamond crystal of the ATR (Attenuated Total Reflection) accessory was cleaned with 70% isopropanol and distilled water after each measurement. The obtained raw spectra were subjected to EMSC (Extended Multiplicative Signal Correction) [26]. Processing of the spectra was performed with The Unscramble X 10.5 (CAMO Software, Oslo, Norway).

#### Statistical Analysis

The growth experiments in the Duetz-MTPS and in the 2.5 L fermenters were carried out in triplicate and duplicate, respectively. The presented results are the mean of the replicates, and the standard deviations are shown as error bars in the figures. Data handling and statistics were performed using the Excel software package (Microsoft Excel 2013, Microsoft Corp., Redmond, WA). ANOVA (α = 0.05) was used to analyze the differences in amino acid compositions, using JPM v.14.1 (SAS, Cary, North Carolina, U.S.) and comparing all pairs using Tukey–Kramer HSD (Honest Significant Difference). The Principal Component Analysis (PCA) was performed using The Unscrambler X, V10.5 (CAMO, Oslo, Norway).

## Results and Discussion

### Characterization of BALI™ and chicken hydrolysate and selection of yeasts

Additional file 1: Table S1 shows that glucose is the main carbon source in the spruce derived-hydrolysate (BALI™, also abbreviated as B in this study). The production and composition of the chicken hydrolysate (CH), prepared using a commercial protease, have been described previously [20]. The protein content of CH, based on the Kjeldahl method, was 65.7 g/L. In the growth experiments described below carbohydrates were dosed based on glucose, whereas the nitrogen source was dosed based on nitrogen content as determined by the Kjeldahl method.

The tested yeast strains were chosen because of their high potential for biotechnological and especially food-related applications. The Thermosacc^®^ Dry-strain is used for industrial ethanol production and after fermentation it is a component in distillers’ grain, which is used as animal feed. *C. jadinii* is known as fodder yeast (usually under its anamorph name *Candida utilis*) and can convert a variety of substrates to high- value biomass [27]. *W. anomalus* is a very robust yeast. It can grow on a variety of different substrates, efficiently degrade phytate and inhibit undesirable microbes, and it has been demonstrated to improve the nutritional value of animal feed [28, 29]. *B. adeninivorans* is a yeast with a very broad substrate spectrum, utilizing, apart from monosaccharides, also for instance aromatic compounds, and degrading phytate. It is also osmo- and thermotolerant and therefore promising for industrial applications [30].

### Growth experiments in microtiter plates

**Fig. 1:**
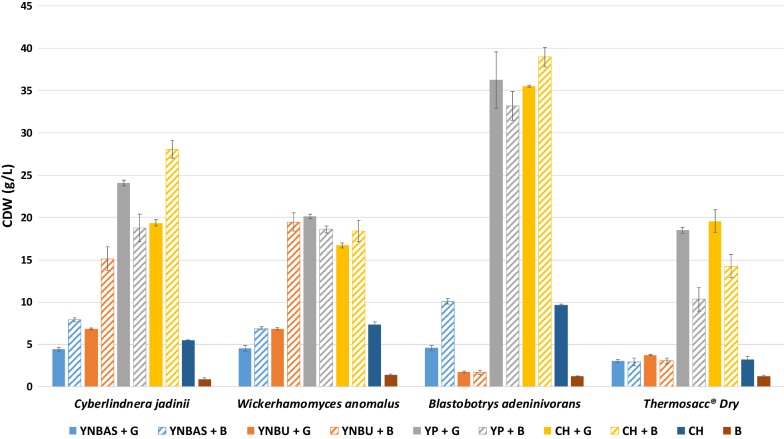
Growth of four yeast strains in the Duetz-MTPS system using 10 different media. The graphs show CDW (g/L) after 24 h cultivation (values are mean ± SD; n = 3), whereas Additional file 1: Figs. S1 and S2 provide more complete growth curves and pH profiles. Conditions: Glucose, 50 g/L; Kjeldahl nitrogen, 5.86 g/L; OD_*init*i*al*_ = 0.5; volume: 2.5 mL; pH_*initial*_= 5.0; incubation at 30 °C with 450 rpm shaking. The pH and pO_2_ were not controlled and several of the apparent differences between media may be due to buffering effects. *YNBAS* yeast nitrogen base without amino acids and with ammonium sulfate, *YNBU* yeast nitrogen base without amino acids and with urea, *YP* yeast extract and meat peptone, *CH* chicken by-products hydrolysate, *B* BALI™ hydrolysate, *G* glucose

A preliminary screening of media in microtiter plates was performed using four different yeasts, *C. jadinii*, *W. anomalus*, *B. adeninivorans* and Thermosacc^®^ Dry. Additional file 1: Figures S1 and S2 show CDW and pH over time, while Fig. 1 shows CDW levels after 24 h. Growth on inorganic nitrogen sources (yeast nitrogen base with ammonium sulfate or with urea) was lower compared to organic nitrogen sources (yeast extract + meat peptone or chicken by-products hydrolysate). In addition, when using inorganic N-sources, the yeasts performed better on BALI sugar than on glucose in several cases. These differences are likely related to differences in buffering capacity of both the nitrogen and the sugar source, as medium acidification occurred rapidly and was more pronounced for media showing low growth (Additional file 1: Figure S2). Importantly, Fig. 1 shows that the chicken by-product hydrolysate (CH) functions as well as the rich YP medium used in this study. When using these rich, well-buffering nitrogen sources, the yeasts performed equally well on glucose (G) and BALI sugar (B) in most cases, but some conspicuous differences were observed. As to the effect of replacing G by B in an otherwise rich medium, results for *C. jadinii* did not provide a consistent picture, whereas the data for Thermosacc^®^ Dry showed a negative impact of B, which could indicate sensitivity of this latter yeast for a compound in B. Of interest, Fig. 1 shows very low growth of Thermosacc^®^ Dry and *B. adeninivorans* on the urea containing medium, indicating that these yeasts lack the enzyme apparatus for urea assimilation.

We considered *B. adeninivorans* interesting as it can use a large variety of substrates as a carbon and nitrogen source [30, 31]. These nitrogen sources include purines, which are abundant in chicken by-products [32]. Indeed, Fig. 1 shows that the by far highest CDW values were reached by *B. adeninivorans* (33–39 g/L) both when using glucose (G) or the BALI sugar solution (B) that contains additional carbohydrates. This translates to cell mass-yields on glucose that are higher than 50%, which in this case would be equal to 25 g/L CDW, which is considered as a typical maximum yield for oxidative growth on sugar [15]. *C. jadinii* and *W. anomalus* produced lower CDW values (between 18 and 28 g/L and 16–20 g/L respectively) on the same media, showing less efficient utilization of YP or CH. Thermosacc^®^ Dry reached 18–20 g/L CDW on YP + G or CH + G, but its growth was lower when using BALI™ as a sugar source (10–14 g/L). In conclusion, these initial growth experiments demonstrated that the combination of the chicken hydrolysate and the BALI™ spruce hydrolysate constitutes a promising growth medium for multiple yeasts.

### Batch fermentations in CH + B and YP + G media

**Fig. 2:**
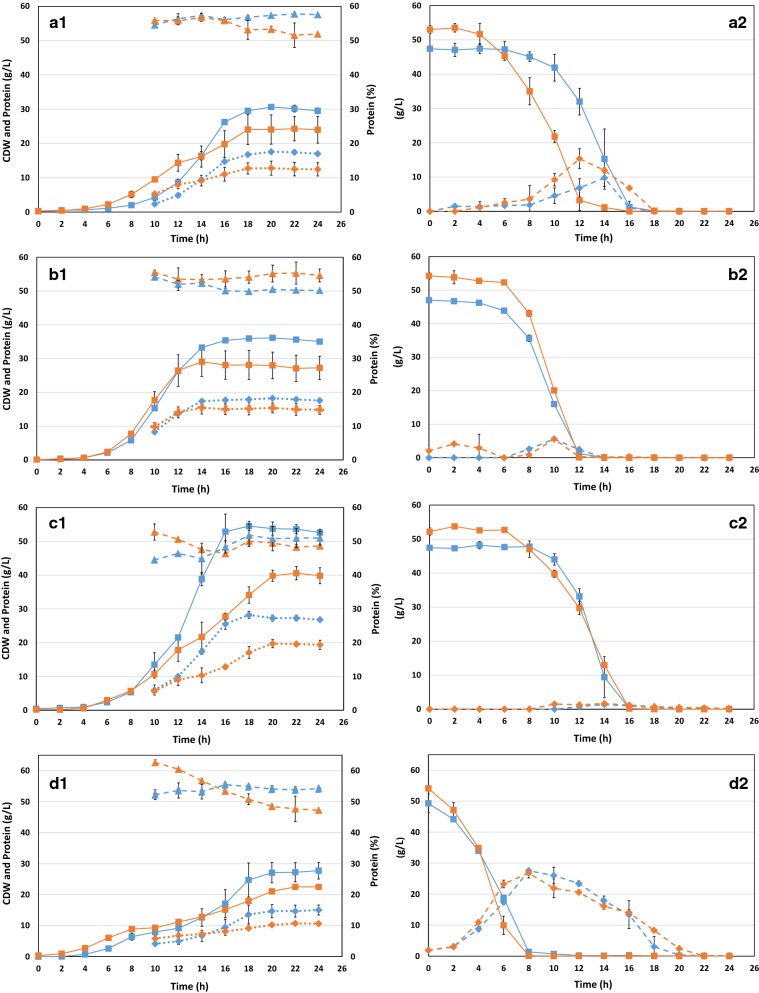
Batch cultivation of four yeast strains on YP + G (orange curves) or CH + B (blue curves) in a 2.5 L benchtop fermenter. The starting volume was 1.5 L and the fermentation lasted 24 h. Panels labeled 1 show accumulation of cells (square symbols, solid lines) and protein (diamond symbols, dotted lines), as well as the protein content of the cells (triangle symbols, dashed lines); panels labeled 2 show glucose (square symbols, solid lines) and ethanol (diamond symbol, dashed lines). **a***C. jadinii*; **b***W. anomalus*; **c***B. adeninivorans*; **d** Thermosacc^®^ Dry. Growth was monitored by measuring the CDW (g/L, square symbols, solid lines) every 2 h. For the samples taken from 10 h and onwards, the protein content (triangle symbol, dashed lines) of the dried cells was measured using the Kjeldahl method (w/w %). The concentration of yeast protein (g/L; diamond symbols, dotted lines) was calculated by multiplying CDW (g/L; square symbols, solid lines) with the protein content (w/w %). Acetic acid and lactic acid production were negligible for all yeasts on both media (results not shown). Values are mean ± SD (n = 2)

Based on the initial experiments described above we carried out a comparative assessment of growth on a rich medium (YP + G) and a rich medium derived from spruce and chicken by-products (CH + B), using fully controlled (pH and pO_2_) benchtop bioreactors. Figure 2 and Table 1 show growth and protein production for the four yeast strains. Table 1 also summarizes yields per gram of sugar, whereas Fig. 2 also shows glucose consumption and ethanol levels. Additional file 1: Figure S3 shows the consumption of free amino nitrogen. Of note, the spruce hydrolysate, B, contains other sugars in addition to glucose (Additional file 1: Table S1) and would a priori be expected to enable higher biomass yields, provided that nitrogen was not limiting and depending on the ability of the yeast strains to ferment sugars other than glucose.

**Table 1:** Data for growth of *C. jadinii*, *W. anomalus*, *B. adeninivorans* and Thermosacc^®^ Dry grown in 2.5 L benchtop fermenters

Medium	*C. jadinii*		*W. anomalus*		*B. adeninivorans*		Thermosacc^®^ Dry	
	YP + G	CH + B	YP + G	CH + B	YP + G	CH + B	YP + G	CH + B
Time (h)	20	20	20	20	20	18	22	20
CDW (g/L)	24.1 ± 4.3	30.6 ± 0.5	28.0 ± 3.9	36.1 ± 0.1	39.8 ± 1.6	54.5 ± 1.4	22.5 ± 0.5	27.1 ± 3.4
Max. growth rate (h^−1^)	0.4 ± 0.1	0.3 ± 0.0	0.6 ± 0.1	0.4 ± 0.0	0.6 ± 0.1	0.4 ± 0.1	0.5 ± 0.0	0.2 ± 0.1
Protein (%)	53.3 ± 0.9	57.4 ± 0.1	55.2 ± 2.5	50.5 ± 0.0	49.5 ± 0.0	51.8 ± 3.3	47.7 ± 4.1	54.1 ± 1.2
Protein (g/L)	12.8 ± 2.1	17.6 ± 0.3	15.4 ± 1.5	18.2 ± 0.0	19.7 ± 1.2	28.2 ± 1.1	10.7 ± 0.6	14.7 ± 2.1
*Y*_X/sugars_		0.49 ± 0.01		0.58 ± 0.01		0.87 ± 0.02		0.42 ± 0.08
*Y*_P/sugars_		0.28 ± 0.00		0.29 ± 0.00		0.45 ± 0.02		0.23 ± 0.05
*Y*_X/glucose_	0.46 ± 0.09	0.65 ± 0.01	0.52 ± 0.06	0.77 ± 0.01	0.76 ± 0.05	1.15 ± 0.03	0.42 ± 0.01	0.55 ± 0.10
*Y*_P/glucose_	0.24 ± 0.04	0.37 ± 0.01	0.28 ± 0.02	0.39 ± 0.00	0.38 ± 0.03	0.60 ± 0.02	0.20 ± 0.01	0.30 ± 0.06

The media used were YP + G or CH + B, and the start volume of the fermentation was 1.5 L. The media contained 5.86 g/L Kjeldahl nitrogen (36.6 g/L protein) and approximately 50 g/L glucose (see t = 0 point in Fig. 2, right panels; note that for B, 50 g/L glucose corresponds to 66.0 g/L total sugars; see Additional file 1: Table S1). The data shown are for the time point (indicated in the Table) at which the concentration of yeast protein (g/L) was the highest. Data for 24 h time points appear in Table 2. Y values refer to yields of CDW (X) or protein (P) per gram of consumed glucose, as measured (right panels in Fig. 2) or per gram of total sugar, as calculated from Additional file 1: Table S1 (for B only). Values are mean ± SD (n = 2)

*Cyberlindnera jadinii* (Fig. 2a) had a longer lag phase on CH + B than on YP + G, but, like all other yeasts (Fig. 2), gave a higher biomass production on the hydrolysate-based medium: 30.6 vs. 24.1 g/L CDW after 20 h (Table 1). The growth continued after glucose depletion, likely due to the aerobic utilization of accumulated ethanol (diauxic shift [15]). Maximal ethanol levels reached 9.7 and 15.4 g/L on CH + B and YP + G, respectively, after about 12 h–14 h. At all measuring points (10–24 h), the protein content of the yeast biomass was above 50% (w/w), and was, at the later time points, higher for the CH + B fermentation, compared to the YP + G fermentation (e.g. 57.4% versus 53.3% at 20 h; Table 1).

*W. anomalus* growth and glucose consumption profiles for both media were very similar until the point of glucose depletion (at 12 h), whereas also in this case maximum biomass yields were higher with CH + B compared to YP + G: 36.1 and 28.0 g/L CDW after 20 h (Fig. 2b, Table 1). Only minor ethanol accumulation was observed with *W. anomalus* (max. 5.6 g/L). The protein content of the yeast biomass was relatively stable over time and was higher on YP + G than on CH + B (e.g. 55.2% versus 50.5% after 20 h; Table 1).

*B. adeninivorans* consumed glucose at similar rates for both media, with negligible ethanol production (less than 1.5 g/L) (Fig. 2c). Final biomass levels were considerably higher for CH + B and the yields were remarkably high: 39.8 g/L on YP + G and 54.5 g/L on CH + B, after 20 and 18 h, respectively (Table 1). These high yields suggest that this yeast is capable of using compounds present in the nitrogen source (YP or CH) and the BALI hydrolysate, which the other yeast strains cannot use. The protein content of yeast biomass was around 50% (w/w) on both media.

Thermosacc^®^ Dry consumed the supplied glucose within 8 h on both media, resulting in undesirable high ethanol concentrations of up to 27 g/L (Fig. 1d). Thermosacc^®^ Dry is a *Saccharomyces cerevisiae* strain specifically developed for ethanol production. Therefore, this strain was expected to be less suitable for SCP production, as was indeed confirmed by the results shown in Fig. 2. Similarly, to *C. jadinii* on YP + G, the diauxic nature of the growth curve is visible for both media: after glucose depletion the accumulated ethanol was consumed during the subsequent 12 h–14 h, resulting in CDW concentrations of 27.1 g/L and 22.5 g/L for CH + B and YP + G, respectively (Table 1). Towards the end of the glucose consumption phase, the protein content for yeast growing on YP + G was very high (62.7%, w/w) but this level was decreased to 47.7% (w/w) after 22 h. For yeast grown on CH + B, the protein content was rather stable, reaching 54.1% (w/w) at 20 h.

Whereas these experiments reveal clear differences between the yeasts, some general trends are visible, which seem to apply to all tested yeasts. While BALI hydrolysates may be considered as a drop-in replacement for other glucose sources, such as starch-derived glucose, these hydrolysates come with the potential benefit of containing an additional 16 g of sugar per 50 g of glucose (Additional file 1: Table S1). Likely as a consequence of this, all yeasts gave higher CDW yields in the CH + B fermentations. Indeed, analysis of the contents of some common monosugars (galactose, arabinose, xylose and mannose) showed that these were largely consumed by the yeasts (Additional file 1: Table S2). Several of the fermentations showed biomass accumulation after the depletion of glucose, which could be due to the extra sugars in fermentations with BALI sugar, but also to consumption of accumulated ethanol, as discussed above for Thermosacc^®^, and to the use of non-carbohydrate carbon sources such as amino acids. The protein content of the yeast cells was 50% or higher in all but one case. The strain, growth medium and growing conditions all may have impact on the content of crude protein. The values obtained here are within protein levels that are considered reasonable in the context of SCP production; protein contents in yeasts normally vary between 45 and 55% [2, 9, 33].

The maximum yields obtained for yeasts growing on carbohydrates (*Y*_x/glucose_) under aerobic conditions typically range between 0.4 and 0.5 g biomass per g of sugar [15]. Similar trends can be observed in our study for all yeasts with the exception of *B. adeninivorans* which showed *Y*_x/glucose_ values of 0.76 and 1.15 g/g for YP + G and CH + B, respectively. Correcting the yield on CH + B for the additional sugars in B, still leaves a yield (*Y*_x/sugars_) of 0.87 g per g of sugar. These high yields confirm the results from the microtiter plate experiments and are likely due to the ability of this yeast to utilize a wide variety of substrates as a carbon and nitrogen source [31]. *C. jadinii* and Thermosacc^®^ Dry had the lowest *Y*_x/sugars_ values when using CH + B as a medium (0.49 g/g and 0.42 g/g, respectively) and these values were very similar to the *Y*_x/glucose_ values obtained when using YP + G (0.46 g/g and 0.42 g/g, respectively). *Y*_x/sugars_ and *Y*_x/glucose_ values for *W. anomalus* grown on CH + B and YP + G were 0.58 g/g and 0.52 g/g respectively, suggesting that this yeast utilized some other compounds next to sugars, albeit not to the same extent as *B. adeninivorans*. Utilization of amino acids both as nitrogen- and carbon sources has been described for a variety of yeast species [34], and is especially efficient in *B. adeninivorans*. This may explain different biomass yields on CH + B.

Protein yields ranged from 0.20 g to 0.29 g per g of glucose (YP + G) or total sugar (CH + B), with the exception of the *B. adeninivorans* fermentations which yielded approximately 0.4 g protein per g of sugar (Table 1). These yields are similar to those described in the literature. For example, in batch fermentations with *C.jadinii*, Lee et al. [35] achieved a high biomass yield of 0.67 g per g of glucose, which, assuming a 50% protein content, corresponds to a protein yield of 0.33 g/g. Also using batch fermentation of *C. jadinii,* Nigam et al. [36] produced SCP from pineapple cannery effluent and obtained a maximum cell biomass and total protein yield of 0.30 and 0.17 g per g of sugar, respectively. The high protein yields obtained with *B. adeninivorans* can likely be explained in part by the abovementioned ability of this yeast to assimilate a wide range of nitrogen-containing carbon sources (proteins, purines etc.). The enhanced growth of this yeast is also reflected in the consumption of free amino nitrogen that was indeed higher for *B. adeninivorans* compared to the other yeast strains (Additional file 1: Figure S3).

### Characterization of yeast biomass

The chemical composition of freeze–dried yeast biomass obtained from the bioreactor cultivations after 24 h of fermentation was determined. Table 2 shows the content of protein, nucleic acids, lipids, carbohydrates and ash. The lipid content of the yeasts (0.4–1.8%) was lower than what is typically found in literature (2–6%) [33]. Lipid accumulation is generally known to be induced by nitrogen starvation [37]. Additional file 1: Figure S3 shows that nitrogen was available during the whole fermentation, in all experiments, which may explain the low levels of lipids. The contents of nucleic acids (2.5–5.8%; Table 2) were also low compared to previous studies (5–12%) [15, 33]. However, in the present experiment, nucleic acid analyses were performed on yeast biomasses obtained after 24 h of batch cultivation. At this point, the yeast cells were probably in a stationary phase (i.e., stable CDW for the last 4 to 10 h before harvesting of cells; Fig. 2). It has previously been observed that microbial cells in stationary phase have a low concentration of nucleic acids, primarily due to a reduction of the RNA content [10].

**Table 2:** Composition of yeasts grown on YP + G or CH + B after 24 h batch fermentation

	*C. jadinii*		*W. anomalus*		*B. adeninivorans*		Thermosacc^®^ Dry	
	YP + G	CH + B	YP + G	CH + B	YP + G	CH + B	YP + G	CH + B
Crude protein^a^	51.9 ± 0.3	57.6 ± 0.2	54.6 ± 1.9	50.2 ± 0.3	48.7 ± 0.5	51.0 ± 2.0	47.3 ± 0.1	54.2 ± 0.5
Nucleic acids	4.4 ± 0.1	4.9 ± 0.2	5.8 ± 0.0	4.1 ± 0.4	2.5 ± 0.2	2.8 ± 0.1	3.1 ± 0.4	2.6 ± 0.0
Crude lipid	0.5 ± 0.2	1.1 ± 0.5	ND	1.2 ± 0.0	0.2 ± 0.1	1.2 ± 0.4	0.4 ± 0.2	1.8 ± 0.1
Est Total Carbohydrates^b^	42.3	35.1	41.2	43.5	45.6	42.3	48.2	37.7
Ash	5.3 ± 0.3	6.2 ± 0.6	4.2 ± 0.4	5.1 ± 0.1	5.5 ± 0.2	5.5 ± 0.5	4.1 ± 0.3	6.3 ± 0.0

The Table shows mean values derived from duplicate fermentations ± standard deviation. Prior to the analysis, yeast cells were washed and freeze–dried. Values are mean ± SD (n = 2)

^a^The protein content equals N x 6.25, which means that non-protein nitrogen is included

^b^The total carbohydrate fraction was estimated as follows: Est Total Carbohydrates = 100 − Crude Protein − Crude lipid − Ash. Nucleic acids are not included here since these are also covered by Kjeldahl nitrogen

Table 3 provides an overview of selected minerals in the yeast biomass. The total amount of minerals was slightly higher for yeast grown on CH + B, especially for Thermosacc^®^ Dry. *W. anomalus* contained the lowest amount of minerals. The most abundant macro elements were potassium, phosphorus, sulfur, and sodium, while most micro elements were found in very low concentrations.

**Table 3:** Macro and trace minerals determined by ICP – MS in freeze dried yeast biomass

	*C. jadinii*		*W. anomalus*		*B. adeninivorans*		Thermosacc^®^ Dry	
Medium	YP + G	CH + B	YP + G	CH + B	YP + G	CH + B	YP + G	CH + B
Macro Minerals (g/kg dry matter)								
Na	1.5 ± 0.1	3.0 ± 0.6	1.1 ± 0.2	1.7 ± 0.8	4.8 ± 0.5	8.8 ± 1.6	1.5 ± 0.4	1.5 ± 0.2
Mg	0.8 ± 0.0	1.0 ± 0.2	0.5 ± 0.0	0.9 ± 0.0	0.6 ± 0.0	1.0 ± 0.0	0.7 ± 0.0	1.1 ± 0.0
P	11.5 ± 0.7	13.0 ± 2.8	7.5 ± 1.0	10.6 ± 0.5	8.2 ± 0.7	8.9 ± 0.6	6.0 ± 0.0	13.0 ± 0.0
S	4.6 ± 0.6	6.4 ± 1.6	3.2 ± 0.4	4.9 ± 1.9	5.4 ± 0.0	8.9 ± 0.4	3.9 ± 0.2	7.6 ± 0.9
K	17.5 ± 0.7	13.5 ± 3.5	11.5 ± 0.7	7.6 ± 3.3	18.0 ± 1.4	11.5 ± 0.7	15.5 ± 0.7	17.0 ± 0.0
Ca	0.1 ± 0.0	0.6 ± 0.1	ND	0.3 ± 0.0	ND	0.7 ± 0.0	ND	3.5 ± 0.4
Cl	0.1 ± 0.1	0.3 ± 0.0	0.3 ± 0.0	0.4 ± 0.0	2.4 ± 0.0	3.9 ± 0.0	0.1 ± 0.1	0.6 ± 0.0
Trace Minerals (mg/kg dry matter)								
Cr	1.7 ± 0.1	0.8 ± 0.3	2.3 ± 0.5	0.5 ± 0.0	3.1 ± 0.4	0.7 ± 0.2	5.5 ± 0.7	0.9 ± 0.4
Mn	1.8 ± 0.0	14 ± 0.0	1.3 ± 0.0	11.6 ± 0.0	1.4 ± 0.0	15 ± 0.0	2.0 ± 0.0	10.8 ± 0.0
Co	0.5 ± 0.0	0.1 ± 0.0	0.4 ± 0.0	ND	0.5 ± 0.0	ND	0.9 ± 0.0	ND
Cu	3.6 ± 2.9	15.5 ± 3.5	9.8 ± 1.7	9.0 ± 0.3	8.8 ± 1.7	1.7 ± 0.8	14.5 ± 3.5	0.9 ± 0.1
Se	ND	0.6 ± 0.0	ND	0.6 ± 0.0	ND	0.7 ± 0.0	ND	0.6 ± 0.0
Al	3.6 ± 1.3	4.1 ± 2.6	3.0 ± 0.4	3.3 ± 0.6	3.7 ± 0.2	4.1 ± 1.1	2.7 ± 0.7	4.3 ± 0.8
Fe	44.0 ± 1.4	255 ± 7.1	34.5 ± 7.8	55.8 ± 2.6	38.5 ± 2.1	160.0 ± 14.1	53.5 ± 6.4	75.0 ± 5.7
Ni	0.8 ± 0.2	0.5 ± 0.3	1.0 ± 0.5	0.3 ± 0.1	1.6 ± 0.4	0.6 ± 0.3	2.6 ± 0.8	0.5 ± 0.5
Zn	105 ± 7.1	165 ± 21.2	71 ± 8.5	146.7 ± 23.6	70.5 ± 2.1	105.0 ± 7.1	91 ± 1.4	150 ± 14.1
Total Elements (g/kg dry matter)	37.3	38.8	25.5	27.4	41.3	44.8	30.7	45.3

Yeasts were grown on YP + G and CH + B media and harvested after 24 h. Values are mean ± SD (n= 2). ND, not detected. No detectable levels of As, Cd, Pb and Br were found in any of the yeast samples

Table 4 presents the amino acid composition of the yeast cells at 18, 20, or 22 h, i.e. at timepoints where the protein concentration (g/L) was at or close to the highest level reached during the batch fermentations. Generally, the amino acid compositions depicted in Table 4 are similar to previously published amino acid compositions of yeast, including characteristic high contents of threonine and lysine and low contents of S-containing amino acids such as methionine and cysteine [10].

**Table 4:** Amino acid composition of yeast biomass obtained after fermentation on YP + G or CH + B medium

	*C.jadinii*		*W. anomalus*		*B. adeninivorans*		Thermosacc dry^®^		Fish meal^c^	Soybean meal^d^
Medium	YP + G	CH + B	YP + G	CH + B	YP + G	CH + B	YP + G	CH + B		
Time (h)^b^	20	20	20	20	20	18	22	20		
EAAs^a^										
Met, M	5.1 ± 0.2^* º^	6.2 ± 0.1^* º^	3.9 ± 0.1^* º^	4.6 ± 0.0^* º^	5.1 ± 0.0^* º^	5.6 ± 0.2^* º^	6.0 ± 0.1^* º^	7.0 ± 0.3^*^	16.1^**º**^	7.7^*^
Thr, T	24.5 ± 0.8	26.3 ± 0.3	21.8 ± 0.8	21.4 ± 0.1	21.3 ± 0.1	23.8 ± 3.0	21.5 ± 0.2	25.1 ± 0.9	25.4	20.2
Val, V	25.0 ± 0.0	29.0 ± 0.3	22.9 ± 0.6^*^	24.7 ± 0.3	20.5 ± 0.2^* º^	21.3 ± 0.8^* º^	25.2 ± 0.6	25.0 ± 0.4	26.4	24.1
Ile, I	21.5 ± 0.3	24.6 ± 0.4	20.6 ± 0.9^*^	21.4 ± 0.0	16.1 ± 0.1^* º^	17.1 ± 0.6^* º^	19.3 ± 0.5^* º^	23.1 ± 0.2	23.7	23.1
Leu, L	32.5 ± 0.8^* º^	38.2 ± 0.6	31 ± 1.4^* º^	32.7 ± 0.2^* º^	33.1 ± 0.3^* º^	33.9 ± 1.3^* º^	29.1 ± 0.4^* º^	35.3 ± 0.3^*^	42	39
His, H	9.2 ± 0^* º^	11.5 ± 0.4	9.2 ± 0.1^* º^	13.6 ± 0.8	8.5 ± 0.3^* º^	13.5 ± 0.3	8.8 ± 0.1^* º^	10.2 ± 0.2^* º^	11.8	13.5
Lys, K	42.2 ± 1.8^**º**^	39.5 ± 0.5^* º^	42.5 ± 0.4	43.1 ± 0	26.8 ± 0.9^* º^	31.1 ± 0.7^*^	32.4 ± 0.7^*^	36.8 ± 0.1^* º^	45.5^**º**^	32.3^*^
Arg, R	31.9 ± 1.7	28.5 ± 0.0	34.6 ± 2.6	20.8 ± 0.2^* º^	21.0 ± 0.3^* º^	23.0 ± 0.9^* º^	24.7 ± 0.4^* º^	23.6 ± 3.6^* º^	35.3	37.4
Phe, F	18.1 ± 0.5^*º^	21.5 ± 0.4^**º**^	17.4 ± 0.8^*º^	19.4 ± 0.5^**º**^	14.0 ± 0.2^*º^	17.3 ± 0.6^*º^	16.6 ± 0.3^*º^	18.9 ± 0^*º^	22.0^**º**^	26.5^*^
Trp, W	6.2 ± 0.1	7.1 ± 0.0^**º**^	5.8 ± 0.3^*^	5.8 ± 0.1^*^	4.9 ± 0.0^*^	6.3 ± 0.1^* º^	5.2 ± 0.0^* º^	6.1 ± 0.1^*^	6.9	6.8
NEAAs^a^										
Asp, D	44.3 ± 1.9^*^	46.7 ± 0.6^* º^	48.4 ± 0.2	42.2 ± 0.7^* º^	36.8 ± 0.5^* º^	40 ± 4.9^* º^	42.1 ± 0.9^* º^	43.8 ± 1.2^* º^	54.7	59.5
Ser, S	23.7 ± 1.1	24.5 ± 0.2	26.7 ± 0.0	23.5 ± 0.0	22.6 ± 0.2^**º**^	21.3 ± 1.1^* º^	21.6 ± 0.1^* º^	22.7 ± 0.0	25.3	25.8
Glu, E	72.1 ± 0.8	76.1 ± 1.8	68.6 ± 0.7^**º**^	64.3 ± 0.9^**º**^	69.1 ± 3.7^**º**^	74.6 ± 10.0	73.5 ± 2.4	73.8 ± 3.1	83.9	92.1
Pro, P	22.0 ± 1.7	18.4 ± 0.6	23 ± 0.4	17.1 ± 0.9	29.2 ± 0.6	22.7 ± 3.7	17.7 ± 0.2	19.8 ± 1.6	23.1	24.1
Gly, G	30.2 ± 3.2^**º**^	23.4 ± 0.6^*^	29.1 ± 1.0^**º**^	20.3 ± 0.0^*^	24.2 ± 0.7	19.1 ± 1.3^*^	21.2 ± 0.6^*^	21.3 ± 0.2^*^	30.8^**º**^	21.6^*^
Tyr, Y	14.6 ± 0.8	18.2 ± 1.2^**º**^	15.0 ± 0.4	17.7 ± 0.6	10.9 ± 0.2^* º^	12.9 ± 0.6	12.7 ± 0.2	15.5 ± 0.1	15.2	14.7
Cys, C	6.2 ± 1.0	5.4 ± 0.2	3.3 ± 0.1^* º^	4.0 ± 0.0^**º**^	3.2 ± 0^* º^	3.5 ± 0.2^* º^	4.1 ± 0.0^**º**^	4.3 ± 0.4^**º**^	5.7	6.9
Ala, A	28.5 ± 0.4^*º^	29.0 ± 0.3^**º**^	25.3 ± 0.4^*^	23.6 ± 0.3^*^	25.2 ± 0.4^*^	26.2 ± 1.8^*^	25.6 ± 0.9^*^	27.8 ± 0.2^*º^	32.6^**º**^	22.4^*^
SUM AA	458.7	475.2	450	421	393.6	414.1	408	440.9	526.4	497.8

Values are mean ± SD (n= 2). ANOVA analyses were performed for each yeast grown on YP + G or CH + B, including a comparison of means for all pairs using Tukey–Kramer HSD. Amino acids levels that differ significantly (α = 0.05) from levels in fish meal or soybean meal are marked by * and º, respectively. EAAs, essential amino acids; NEAAs, non-essential amino acids

^a^All values are in g/kg of dry matter

^b^Time point (h) during batch fermentations where the yeast protein concentration (g/L) was at or close to its maximum

^c^The content of amino acids in fish meal (except tryptophan) was taken from Ref. [38]; the value for tryptophan comes from Ref. [39]

^d^The content of amino acids in soybean meal was taken from Ref. [40]

Since yeast potentially may be used as an ingredient in fish feed [10], we compared the amino acid composition of the four yeast strains with the amino acid compositions of fish- and soybean meals. The measured sums of amino acids varied between 393.6 and 475.2 g/kg dry matter for the four yeast strains (Table 4). The total amino acid contents of a standard fish meal and soybean meal were determined to be 526.4 and 497.8 g/kg dry matter, respectively (Table 4). These latter values are slightly higher than those observed for the yeasts, but in some cases, the difference is small: *C. jadinii* on CH + B gave 475.2 g/kg, versus 497.8 g/kg for soybean meal. The total amounts of amino acids were slightly higher when using CH + B as a medium, except for *W. anomalus,* which is in accordance with the Kjeldahl-based protein concentrations (Table 2).

A PCA analysis (Additional file 1: Figure S4) showed that the fermentation medium had limited effects on the amino acid composition of the four yeast strains. For example, for all four yeasts, the difference between the two media was smaller than the difference between the two reference materials. The PCA plot also shows that the amino acid compositions of *C. jadinii* and *W. anomalus* are most similar to the composition of fish meal. Table 4 shows the results of ANOVA analysis done to detect differences at the individual amino acid level between the yeasts and the two reference protein sources. Among other things, the Table shows that the differences between fish and soybean meal primarily concern Met, Lys, Gly, Ala and Phe.

**Fig. 3:**
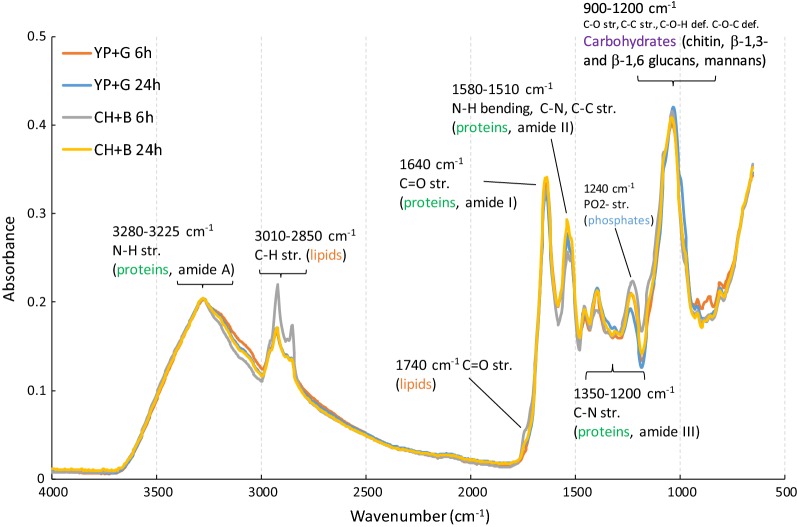
FTIR spectra of *C. jadinii* biomass obtained after 6 h or 24 h growth on YP + G or CH + B. The main vibration bands (and modes), with corresponding bio-macromolecular groups, are indicated

FTIR spectra of freeze–dried yeast cells showed expected features and were similar for all four yeasts, independent of the medium used (Fig. 3 and Additional file 1: Figure S5). Figure 3 shows spectra for *C. jadinii*, grown on YP + G or CH + B, and sampled at 6 h and 24 h; spectra for the other yeasts are provided in Additional file 1: Fig. S5. The spectra are dominated by N–H, C=O, C–N, C–C stretching and N-H bending vibrations from the amide groups of proteins (3280–3225, 1640, 1580–1510, 1350–1200 cm^−1^) and by C–O, C–C, C–O–H and C–O–C stretching and deformation vibrations from carbohydrates (900–1200 cm^−1^). Minor contributions of C–H and C=O stretching vibrations from lipids (3010–2850, 1740 cm^−1^) and PO_2_^−^ stretching vibrations from nucleic acids and phospholipids (1240 cm^−1^) are also visible. There are no major differences between the two sampling time points (Fig. 3 and Additional file 1: Figure S5) except that the early samples (6 h) show a stronger lipid signal for fermentations on CH+B medium, which is probably due to the soluble lipids present in CH.

## Conclusions

In conclusion, this proof-of-concept study demonstrates a possible way of upgrading low value industrial side streams to yeast biomass that can be used as a high quality feed ingredient. Bioreactor experiments showed that yeast biomass and protein production values on BALI spruce hydrolysate + chicken by-products hydrolysate were similar or better than when using a traditional glucose + yeast extract medium. The protein content of the yeast biomass was high (around 50 w/w %), while the nucleic acid content was very low; these are both favorable features of SCP. Importantly, the amino acid profiles of the yeasts were similar to those of fishmeal. *B. adeninivorans* is a promising candidate for single cell protein production due to its exceptional ability to utilize a wide range of compounds for growth without producing ethanol. While *C. jadinii* is well established as high-potential SCP with beneficial functional properties [9], less is known for *B. adeninivorans*. Further studies on the performance of *B. adeninivorans* as SCP in diets for animals and fish are needed, and considering the results presented here, of major interest.

Further research is needed to develop an economically viable yeast production process based on industrial side streams as substrates. The cost of the chicken by-product hydrolysates could be decreased by applying only endogenous enzymes for hydrolysis. It may also be possible to replace part of the presumably “rich” chicken by-product hydrolysates by cheap inorganic nitrogen sources. Repeated batch or continuous fermentation modes are known to be most suitable for microbial biomass production processes [15] and should be explored in future work. Downstream processing of the yeast biomass (drying steps, possible mechanical or autolytic lysis of the cells, formulation) also needs to be addressed since such processing will affect nutritional value. For example, the yeasts may be used as whole cells, or they may be subjected to a cell-wall destructing treatment that may increase digestibility. Other processing steps, such as removal of nucleic acids, could also be envisaged. However, for use in diets for salmon, this will probably not be necessary as the uricolytic pathway in salmon can handle very high dietary levels of nucleic acids [41].

Importantly, besides providing protein, minerals and vitamins, yeasts in feed may have positive health effects as a result of the presence of bioactive and immunostimulating compounds such as ß-glucans and α-mannans [13]. Therefore, further detailed compositional analysis of the yeast cell walls is of interest. Finally, fish feeding experiments should be carried out in order to assess the true potential of these yeast as a feed ingredient [10].

## Supplementary information

**Additional file 1.** Additional figures and tables.

## Data Availability

All data generated or analyzed during this study are included in this published article (and its additional files).

## Abbreviations

SCP: Single cell protein
YNBAS: Yeast nitrogen base without amino acids with ammonium sulfate
YNBU: Yeast nitrogen base without amino acids with urea
YP: Yeast extract and meat peptone
CH: Chicken by-products hydrolysate
G: Glucose
B: BALI™ spruce hydrolysate
CDW: Cell dry weight
HPLC: High-performance liquid chromatography
ICS: Ion chromatography system
ICP-MS: Inductively coupled plasma mass spectrometry
FTIR: Fourier-transform infrared spectroscopy
PCA: Principal component analysis
AA: Amino acid
EAAs: Essential amino acid
NEAA: Non-essential amino acid
ANOVA: Analysis of variance
MTPS: Microtiter plate system
*Y*_X/sugars_: Yield, g dry yeast per g sugar fed (g/g)
*Y*_P/sugars_: Yield, g dry yeast protein per g sugar fed (g/g)
*Y*_X/glucose_: Yield, g dry yeast per g consumed glucose (g/g)
*Y*_P/glucose_: Yield, g dry yeast protein per g consumed glucose (g/g)

## References

[1] United Nations (2017). World Population Prospects The 2017 revision key findings and advance tables. World Popul Prospect.

[2] Ritala A, Häkkinen ST, Toivari M, Wiebe MG (2017). Single cell protein-state-of-the-art, industrial landscape and patents 2001–2016. Front Microbiol..

[3] Pimentel D, Pimentel M (2018). Sustainability of meat-based and plant-based diets and the environment. Am J Clin Nutr.

[4] FAO. The State of World Fisheries and Aquaculture 2016 (SOFIA). Contributing for food security and nutrition for all. Rome; 2016.

[5] FAO. Fisheries and Aquaculture Circular No. 1089. Economic analysis of supply and demand for food up to 2030—special focus on fish and fishery products. Vol. 1089, FAO Fisheries and Aquaculture Circular. 2014. p. 48–60.

[6] Fry JP, Mailloux NA, Love DC, Milli MC, Cao L (2018). Feed conversion efficiency in aquaculture : do we measure it correctly ?. Environ Res Lett..

[7] FAO Fisheries Circular No. 975. Use of Fishmeal and Fish Oil in Aquafeeds—further thoughts on the fishmeal trap. FAO Fisheries Circular No. 975. 2002.

[8] Naylor RL, Hardy RW, Bureau DP, Chiu A, Elliott M, Farrell AP (2009). Feeding aquaculture in an era of finite resources. Proc Natl Acad Sci..

[9] Øverland M, Skrede A (2017). Yeast derived from lignocellulosic biomass as a sustainable feed resource for use in aquaculture. J Sci Food Agric.

[10] Øverland M, Karlsson A, Mydland LT, Romarheim OH, Skrede A (2013). Evaluation of Candida utilis, Kluyveromyces marxianus and Saccharomyces cerevisiae yeasts as protein sources in diets for Atlantic salmon (Salmon salar). Aquaculture.

[11] Kogan G., Kocher A. (2007). Role of yeast cell wall polysaccharides in pig nutrition and health protection. Livestock Science.

[12] Jacob JP, Pescatore AJ (2014). Barley β-glucan in poultry diets. Ann Transl Med..

[13] Meena DK, Das P, Kumar S, Mandal SC, Prusty AK, Singh SK (2013). Beta-glucan: An ideal immunostimulant in aquaculture (a review). Fish Physiol Biochem..

[14] Stanbury PF, Whitaker A, Haller SJ (2000). Principles of fermentation technology.

[15] Ugalde UO, Castrillo JI (2002). Single cell proteins from fungi and yeasts. Appl Mycol Biotechnol..

[16] Rødsrud G, Lersch M, Sjöde A (2012). History and future of world’s most advanced biorefinery in operation. Biomass Bioenergy.

[17] Müller G, Chylenski P, Bissaro B, Eijsink VGH, Horn SJ (2018). Biotechnology for Biofuels The impact of hydrogen peroxide supply on LPMO activity and overall saccharification efficiency of a commercial cellulase cocktail. Biotechnol Biofuels.

[18] Chylenski P, Forsberg Z, Ståhlberg J, Várnai A, Lersch M, Bengtsson O (2017). Development of minimal enzyme cocktails for hydrolysis of sulfite-pulped lignocellulosic biomass. J Biotechnol.

[19] Chylenski P, Petrović DM, Müller G, Dahlström M, Bengtsson O, Lersch M (2017). Biotechnology for Biofuels Enzymatic degradation of sulfite—pulped softwoods and the role of LPMOs. Biotechnol Biofuels.

[20] Lapeña D, Vuoristo KS, Kosa G, Horn SJ, Eijsink VGH (2018). Comparative assessment of enzymatic hydrolysis for valorization of different protein-rich industrial byproducts. J Agric Food Chem.

[21] Abernathy D, Spedding G, Starcher B (2009). Analysis of protein and total usable nitrogen in beer and wine using a microwell ninhydrin assay. J Inst Brew.

[22] Zhao Y, Xiang S, Dai X, Yang K (2013). A simplified diphenylamine colorimetric method for growth quantification. Appl Microbiol Biotechnol.

[23] Sun H, Ge X, Lv Y, Wang A (2012). Application of accelerated solvent extraction in the analysis of organic contaminants, bioactive and nutritional compounds in food and feed. J Chromatogr A.

[24] Mesko MF, Mello PA, Bizzi CA, Dressler VL, Knapp G, Flores ÉMM (2010). Iodine determination in food by inductively coupled plasma mass spectrometry after digestion by microwave-induced combustion. Anal Bioanal Chem.

[25] Sluiter A, Hames B, Ruiz R, Scarlata C, Sluiter J, Templeton D. Determination of ash in biomass: laboratory analytical procedure (LAP). NREL/TP-510-42622. 2008.

[26] Kohler A, Kirschner C, Oust A, Martens H (2006). EMSC as a tool for separation and characterization of physical and chemical information in fourier transform infrared microscopy images of cryo-sections of beef loin. Appl Spectrosc.

[27] Tomita Y, Ikeo K, Tamakawa H, Gojobori T, Ikushima S (2012). Genome and transcriptome analysis of the food-yeast *Candida utilis*. PLoS ONE..

[28] Welin JB, Lyberg K, Passoth V, Olstorpe M (2015). Combined moist airtight storage and feed fermentation of barley by the yeast Wickerhamomyces anomalus and a lactic acid bacteria consortium. Front Plant Sci..

[29] Olstorpe M, Passoth V (2011). Pichia anomala in grain biopreservation. Antonie Van Leeuwenhoek.

[30] Malak A, Baronian K, Kunze G (2016). Blastobotrys (Arxula) adeninivorans: a promising alternative yeast for biotechnology and basic research. Yeast.

[31] Middelhoven WJ, de Jong IM, de Winter M (1991). Arxula adeninivorans, a yeast assimilating many nitrogenous and aromatic compounds. Antonie Van Leeuwenhoek.

[32] Spalvins K, Ivanovs K, Blumberga D (2018). Single cell protein production from waste biomass: review of various agricultural by-products. Agron Res..

[33] Bajpai P (2017). Single cell protein production from lignocellulosic biomass.

[34] Freese S, Vogts T, Speer F, Schäfer B, Passoth V, Klinner U (2011). C- and N-catabolic utilization of tricarboxylic acid cycle-related amino acids by Scheffersomyces stipitis and other yeasts. Yeast.

[35] Lee B, Kim JK (2001). Production of Candida utilis biomass on molasses in different culture types. Aquac Eng.

[36] Nigam JN (1998). Single cell protein from pineapple cannery effluent. World J Microbiol Biotechnol.

[37] Chopra J, Sen R (2018). Process optimization involving critical evaluation of oxygen transfer, oxygen uptake and nitrogen limitation for enhanced biomass and lipid production by oleaginous yeast for biofuel application. Bioprocess Biosyst Eng.

[38] Hansen JØ, Penn M, Øverland M, Shearer KD, Krogdahl Å, Mydland LT (2010). High inclusion of partially deshelled and whole krill meals in diets for Atlantic salmon (Salmo salar). Aquaculture.

[39] Skrede A, Berge G, Storebakken T, Herstad O, Aarstad KG, Sundstøl F (1998). Digestibility of bacterial protein grown on natural gas in mink, pigs, chicken and Atlantic salmon. Anim Feed Sci Technol.

[40] Sriperm N, Pesti GM, Tillman PB (2011). Evaluation of the fixed nitrogen-to-protein (N:P) conversion factor (6.25) versus ingredient specific N: P conversion factors in feedstuffs. J Sci Food Agric..

[41] Andersen Ø, Aas TS, Skugor S, Takle H, Van Nes S (2006). Purine-induced expression of urate oxidase and enzyme activity in Atlantic salmon (Salmo salar) Cloning of urate oxidase liver cDNA from three teleost species and the African lungfish *Protopterus annectens*. FEBS J.

