# Supplementary material for: Production and characterization of yeasts grown on media composed of spruce-derived sugars and protein hydrolysates from chicken by-products
Source: Microb Cell Fact. 2020 Feb 3;19:19. doi: 10.1186/s12934-020-1287-6 (PMC6998301; doi:10.1186/s12934-020-1287-6)
Supplement: Supplementary file 1 — Additional file 1. Additional figures and tables. [file 12934_2020_1287_MOESM1_ESM.docx]

**Additional Material**

**Production and characterization of yeasts grown on media composed of spruce-derived sugars and protein hydrolysates from chicken by-products**

[David Lapeña^1^,](https://no.linkedin.com/in/davidlapenagomez) Gergely Kosa^1^, Line D. Hansen^1^, Liv T. Mydland^2^, Volkmar Passoth^3^, Svein J. Horn^1^, and Vincent G.H. Eijsink^1*^

*^1^ Faculty of Chemistry, Biotechnology and Food Science, Norwegian University of Life Sciences (NMBU), P.O. Box 5003, N-1432 Ås, Norway*

*^2^ Department of Animal and Aquacultural Sciences, Norwegian University of Life Sciences, P.O. Box 5003, N-1432 Ås, Norway*

*^3^ Department of Molecular Sciences, Swedish University of Agricultural Sciences, P.O. Box 7015, S-75007 Uppsala, Sweden*

*Corresponding author:

Telephone: +4767232463

Email: vincent.eijsink@nmbu.no

Email:

David Lapeña: david.lapna.gomez@nmbu.no

Svein J. Horn: svein.horn@nmbu.no

Gergely Kosa: gergely.kosa@nmbu.no

Line D. Hansen: line.degn.hansen@nmbu.no

Liv T. Mydland: liv.mydland@nmbu.no

Volkmar Passoth: volkmar.passoth@slu.se

**Contents:**

Figure S1. Cell dry weight (CDW) in microtiter plate growth experiments

Figure S2. pH in microtiter plate-growth experiments

Figure S3. Free amino nitrogen (FAN) in 1.5 L batch fermentations

Figure S4. Principal component analysis (PCA) of amino acid compositions

Figure S5. FTIR spectra of freeze-dried yeasts

Table S1. Composition of the BALI^TM^ spruce hydrolysate.

Table S2. Monosaccharide analysis for 1.5L batch fermentations.

**Figure S1.**  **Cell dry weight (CDW) in microtiter plate experiments.** Microtiter plate experiments were carried out to assess the growth of four yeast strains on 10 different media. Conditions: 24h cultivation in the Duetz-system with deepwell microtiter plates (2.5 mL/11mL) for 24h, initial pH: 5.0, shaking speed: 450 rpm. The CDW values are means ± SD (n = 3). The media contained 5.86 g/L Kjeldahl nitrogen (36.63 g/L protein) and 50 g/L glucose (note that for B, 50 g/L glucose corresponds to 66 g/L total sugars; see Table S1). Abbreviations: YNBAS, yeast nitrogen base without amino acids and with ammonium sulfate; YNBU, yeast nitrogen base without amino acids and with urea; YP, yeast extract and meat peptone; CH, chicken by-products hydrolysate; B, BALI^TM^ hydrolysate; G, glucose.

**Figure S2. pH in microtiter plate growth experiments.** The graphs show the pH during the growth experiments depicted in Figure S1. Values are means ± SD (n = 3). Abbreviations: YNBAS, yeast nitrogen base without amino acids and with ammonium sulfate; YNBU, yeast nitrogen base without amino acids and with urea; YP, yeast extract and meat peptone; CH, chicken by-products hydrolysate; B, BALI^TM^ hydrolysate; G, glucose.

**Figure S3.** **Free amino nitrogen (FAN) in 1.5 L batch fermentations.** The graphs show the level of free amino nitrogen (g/L) during growth of four yeast strains in 2.5 L total volume benchtop bioreactors. Values are mean ± SD (n = 2). Abbreviations: YP, yeast extract and meat peptone; CH, chicken by-products hydrolysate; B, BALI^TM^ hydrolysate; G, glucose.

**Figure S4.** Principal component analysis (PCA) of amino acid compositions. a) score plot and b) loading plot.

**Figure S5. FTIR spectra of freeze-dried yeasts.** The graphs show FTIR spectra of freeze-dried *W. anomalus*, *B. adeninivorans* and Thermosacc® Dry cells harvested after 6h and 24h cultivation in 2.5 L total volume benchtop bioreactors, using the indicated media. Spectra for C. *jadinii* are provided in the main manuscript. The obtained raw spectra were subjected to EMSC (Extended Multiplicative Signal Correction). Abbreviations: YP, yeast extract and meat peptone; CH, chicken by-products hydrolysate; B, BALI^TM^ hydrolysate; G, glucose.

**Table S1.** **Composition of the BALI^TM^ spruce hydrolysate.**

|  |  |
| --- | --- |
| Content | BALI^TM^ |
| Dry matter (%, w/w) | 62.3 |
| Density (kg/L) | 1.29 |
| Total sugars (% DM) | 90.0 |
| Glucose | 68.2 |
| Xylose | 5.4 |
| Mannose | 6.5 |
| Other sugars^a^ | 9.9 |
| Acids^b^ (% DM) | 1.2 |
| Glycerol (% DM) | 0.2 |
| Lignin (% DM) | 5.3 |

^a^Sum of fructose, arabinose, galactose, gentobiose and cellobiose. ^b^Sum of lactic, formic and acetic acid.

**Table S2. Monosaccharide analysis for 1.5L batch fermentations.** The table shows the content of selected monosaccharides (g/L) in the fermentation medium (CH + B, which is chicken hydrolysate + BALI sugar) before (0 h) and after (24 h) fermentation. Values are means ± SD (n = 3). ND, Not detectable; -, not determined.

| **Time points (h)** | **Yeast strain** | **Galactose** | **Arabinose** | **Xylose** | **Mannose** |
| --- | --- | --- | --- | --- | --- |
| **0** |  | **1.0**^1^ | **0.6**^1^ | **4.0**^1^ | **4.8**^1^ |
| **24** | ***C. jadinii*** | **0.06 ± 0.01** | **0.02 ± 0** | **0.01 ± 0.01** | **ND** |
|  | ***W. anomalus*** | **ND** | **ND** | **ND** | **ND** |
|  | 1. ***adeninivorans*** | **ND** | **ND** | **ND** | **ND** |
|  | **Thermosacc Dry®** | **0.02 ± 0** | **ND** | **0.29 ± 0.12** | **ND** |

^1^ Calculated based on Table S1.
